# Melatonin Promotes Muscle Growth and Redirects Fat Deposition in Cashmere Goats via Gut Microbiota Modulation and Enhanced Antioxidant Capacity

**DOI:** 10.3390/antiox14060645

**Published:** 2025-05-27

**Authors:** Di Han, Zibin Zheng, Zhenyu Su, Xianliu Wang, Shiwei Ding, Chunyan Wang, Liwen He, Wei Zhang

**Affiliations:** 1 State Key Laboratory of Animal Nutrition and Feeding, College of Animal Science and Technology, China Agricultural University, Beijing 100193, China; handi790302@163.com (D.H.); zhengzibin@cau.edu.cn (Z.Z.); suzhenyu@cau.edu.cn (Z.S.); wangxl202203@163.com (X.W.); helw@cau.edu.cn (L.H.); 2 Liaoning Agricultural and Rural Development Service Center, Shenyang 111000, China; daweige317@126.com (S.D.); wangchy1999@163.com (C.W.)

**Keywords:** skeletal muscle hypertrophy, lipid partitioning, antioxidant enzymes, gastrointestinal microbiota, meat quality

## Abstract

Liaoning cashmere goats is a dual-purpose breed valued for premium cashmere fiber and meat yields, and there is currently a lack of optimized strategies for meat quality, including skeletal muscle development and lipid partitioning. This investigation systematically examines how melatonin administration modulates gastrointestinal microbiota and antioxidant capacity to concurrently enhance skeletal muscle hypertrophy and redirect lipid deposition patterns, ultimately improving meat quality and carcass traits in Liaoning cashmere goats. Thirty female half-sibling kids were randomized into control and melatonin-treated groups (2 mg/kg live weight with subcutaneous implants). Postmortem analyses at 8 months assessed carcass traits, meat quality, muscle histology, plasma metabolites, and gut microbiota (16S rRNA sequencing). Melatonin supplementation decreased visceral adiposity (perirenal, omental, and mesenteric fat depots with a *p* < 0.05) while inducing muscle fiber hypertrophy (*longissimus thoracis et lumborum* (LTL) and biceps femoris (BF) with *p* < 0.05). The melatonin-treated group demonstrated elevated postmortem pH_24_h values, attenuated muscle drip loss, enhanced intramuscular protein deposition, and improved systemic antioxidant status (characterized by increased catalase and glutathione levels with concomitant reduction in malondialdehyde with *p* < 0.05). Melatonin reshaped gut microbiota, increasing α-diversity (*p* < 0.05) and enriching beneficial genera (*Prevotella*, *Romboutsia*, and *Akkermansia*), while suppressing lipogenic *Desulfovibrio* populations, and concomitant with improved intestinal morphology as evidenced by elevated villus height-to-crypt depth ratios. These findings establish that melatonin-mediated gastrointestinal microbiota remodeling drives anabolic muscle protein synthesis while optimizing fat deposition, providing a scientifically grounded strategy to enhance meat quality.

## 1. Introduction

Cashmere goats, particularly the Liaoning cashmere goat breed, are globally recognized for their economic importance owing to their high-quality cashmere fiber and meat production [1]. While substantial research has focused on improving cashmere yield through nutritional interventions [2], strategies to optimize meat productivity and quality in these dual-purpose livestock remain underexplored. Skeletal muscle constitutes 45–60% of somatic mass in mature vertebrates [3], orchestrating essential locomotor and metabolic functions and acting as the principal source of animal-derived protein for human diets. Myofiber histomorphometric parameters (fiber number and cross-sectional area) directly modulate carcass yield and meat quality attributes, ultimately governing the commercial valuation of livestock species [3,4]. Skeletal muscle growth, characterized by myofiber hypertrophy and connective tissue remodeling, directly determines meat yield and textural properties such as tenderness and water-holding capacity [5]. Notably, myofiber diameter is a key histological indicator of muscle hypertrophy, with broader fibers correlating with increased muscle mass and improved postmortem meat quality in ruminants [6]. However, excessive intramuscular fat deposition and connective tissue accumulation in goats often compromise these attributes, necessitating strategies to enhance lean muscle growth while maintaining optimal meat biochemistry [7].

Melatonin, a pleiotropic regulator with free radical-scavenging and antioxidant properties, governs cellular proliferation and differentiation, while modulating muscle metabolism and redox homeostasis in livestock [8,9,10]. Murine models reveal that melatonin pretreatment attenuates PI3K/Akt pathway activation, suppresses muscle RING-finger protein-1 (MuRF1) upregulation, and mitigates sepsis-induced muscle atrophy in cecal ligation and puncture (CLP) models, as well as lipopolysaccharide (LPS)-treated C2C12 myotubes [11]. In livestock production, melatonin administration enhances skeletal muscle growth via myofiber hypertrophy, improves mitochondrial bioenergetics, and reduces intramuscular adipogenesis in weaned piglets [9]. Ovine studies further indicate that melatonin implantation elevates circulating anabolic hormones (growth hormone and testosterone), immunoglobulins (immunoglobulin A (IgA), immunoglobulin M (IgM)), and albumin, while increasing the cross-sectional area of muscle fibers and modulating adipocyte morphology [8]. Despite these interspecies’ mechanistic advances, the tissue-specific effects of melatonin on myofiber ultrastructure, extracellular matrix composition, and their integrative relationship with the carcass characteristics in goats remain undefined.

The gastrointestinal microbiota of cashmere goats represents a dynamic symbiotic consortium essential for nutrient assimilation, immunoregulation, and metabolic homeostasis [12,13]. This microbial ecosystem engages in complex mutualistic interactions with the host digestive system, facilitating the enzymatic hydrolysis of dietary substrates [14]. Carbohydrate fermentation generates short-chain fatty acids (SCFAs), which serve as both energy substrates for the host and signaling molecules that modulate the activity of digestive hydrolases (cellulases, proteases, and lipases), thereby enhancing protein and energy utilization efficiency [15,16]. Amino acid-catabolizing bacteria, ubiquitously colonizing mammalian gastrointestinal tracts, microbial proteolysis, and de novo synthesis establish a bidirectional amino acid flux during proteolytic metabolism: luminal and host-derived proteins supply microorganisms with substrates for microbial protein synthesis and ATP generation, while microbial metabolic byproducts contribute to the host’s amino acid pool for anabolic processes [17,18]. Strategic modulation of the gastrointestinal microbiota may optimize amino acid bioavailability and myofibrillar protein synthesis, thereby improving meat quality metrics such as tenderness, water-holding capacity, and intramuscular fat distribution, ultimately enhancing meat quality.

This study hypothesized that melatonin supplementation may remodel gastrointestinal microbiota optimizes amino acid bioavailability and myofibrillar protein synthesis, thereby driving myofiber diameter expansion and skeletal muscle hypertrophy. By integrating histomorphometric analysis of key muscles (*longissimus thoracis et lumborum* (LTL) Gluteus (GL), and biceps femoris (BF)) with 16S rRNA sequencing of gastrointestinal microbiota, we aimed to elucidate the multifactorial mechanisms underlying melatonin’s effects on muscle growth and meat production efficiency. The findings provide novel insights into melatonin’s role as a regulator of myofiber development in ruminants, offering practical strategies to optimize the meat yield and quality of cashmere goat.

## 2. Materials and Methods

### 2.1. Animal Treatment

Thirty female half-sibling Liaoning cashmere goat kids (*Capra hircus*), aged 10 ± 5 days, were randomly assigned to two groups (*n* = 15 per group). Mean birth weights were 3.53 ± 0.29 kg (control group) and 3.51 ± 0.15 kg (melatonin group). The melatonin-treated group received subcutaneous implants at the base of the left ear at 15, 75, and 135 days of age, and melatonin implants (Kangtai Biotechnology Co., Ltd., Beijing, China) were purchased commercially. Melatonin dosage (2 mg/kg live weight) was determined from our previous study [19]. Implants released melatonin over two months, maintaining efficacy until approximately 180 days of age. Kids were housed at a commercial breeding farm in Liaoyang City, Liaoning Province, China (41°16′ N, 123°12′ E), under standardized feeding and management conditions. The dietary nutrient composition is detailed in Table 1.

Ten does approximating the mean body weight (27.24 ± 1.17 kg) were chosen for the slaughter trial at 8 months old. Animals fasted overnight and euthanized. Height at withers was measured vertically from the ground to the highest point of the shoulder blades. Body length was recorded from the scapula to the ischial tuberosity, and chest circumference was measured at the posterior edge of the scapula using a flexible tape. Blood samples were collected intravenously. Carcass metrics included eye muscle area, GR value (tissue depth between the 12th and 13th ribs and 11 cm lateral to the dorsal midline), and backfat thickness (subcutaneous fat at the 12th–13th thoracic vertebrae junction), and all the measurements were on one side of the carcass. Head, hoof, heart, liver, spleen, lungs, kidneys, ovarian, and rumen were weighed. Organ index (%) = (organ weight/slaughter weight) × 100. Muscle tissues (LTL, GL, and BF) were collected and weighed, muscle quality, crude protein content (CP), and crude fat content (ether extract, EE) were determined, and all the muscle samples were on one side of the carcass. Adipose tissue (perirenal fat, greater omental, and mesentery fat) was taken and weighed. Rumen fluid and intestinal content (ileum, cecum, colon, and rectum) were sampled for 16S rRNA sequencing. The muscle and gastrointestinal tract tissues were fixed in 4% paraformaldehyde, sectioned, and stained with hematoxylin-eosin (H&E) for histological evaluation.

### 2.2. Carcass Trait Analysis

Hot carcass weight (post-evisceration, excluding skin, head, extremities, and viscera) was recorded 30 min postmortem. Dressing percentage was calculated as the following: dressing percentage (%) = (dressed carcass weight/antemortem live weight) × 100. A transverse section of the LTL at the thoracolumbar junction was outlined on acid-resistant sulfite paper (Whatman Grade 597, Cytiva, Shanghai, China). The tracing was overlaid with a calibrated 1 × 1 cm grid (Fisher Scientific™ with a precision ± 0.05 cm^2^, Beijing, China) for planimetric analysis. Cross-sectional area determination followed standardized grid-counting protocols: complete squares were enumerated, while partial squares at tissue margins were resolved using a cardinal-direction exclusion principle (upper and left boundaries included and lower and right boundaries excluded).

### 2.3. Meat Quality Evaluation

Postmortem pH was measured in triplicate at 45 min (pH_45_min) and 24 h (pH_24_h) using a pH meter (Testo 205, Testo Ltd., Titisee-Neustadt, Germany) inserted into three sites of trimmed LTL, BF, and GL muscles (5 cm × 1 cm × 0.5 cm) [20]. Meat color parameters including lightness (L*), redness (a*), and yellowness (b*) were measured on the new cutting surface of the samples using a TC-P2A chromameter (Aoike Optoelectronic, Beijing, China) with an 5 mm aperture, D65 illuminant, and 10° observer angle.

Fresh muscle specimens (approximately 30 ± 2 g) were prepared within 1 h post-excision using a standardized drip loss protocol. Samples were vertically suspended in sealed polyethylene containers (15 cm diameter × 20 cm height) through stainless steel meat hooks (2 mm diameter), ensuring complete avoidance of compressive contact. The assembly was maintained at 2–4 °C under 95% relative humidity for 24 h. Drip loss (%) was determined as the following: Drip loss (%) = [(W_1_ − W_2_)/ W1] × 100, where W_1_ and W_2_ represent pre- and post-storage weights.

Samples were vacuum-sealed in polyethylene bags (model: VAC-100; thickness: 80 µm; vapor permeability: 3 g/m^2^/24 h at 25 °C; and Beijing Saizhenbo Technology Co., Ltd., Beijing, China) to minimize moisture loss. Cooking yield (%) was calculated as the following: Cooking yield (%) = [(W_1_ − W_2_)/ W_1_] × 100, where W_1_ and W_2_ represent pre- and post- heating (75 °C water bath and 45 min). Triplicate measurements were averaged for analysis [20].

Shear force (N) was measured perpendicular to fiber orientation using a texture analyzer (C-LM3, Northeast Agricultural University, Harbin, China) following standardized protocols. LTL samples were equilibrated in a precision-controlled water bath at 72 °C until reaching a core temperature of 70 °C. Following thermal stabilization at ambient temperature via hydro-cooling, cylindrical cores (1.27 cm diameter) were excised from the muscle tissue using a rotary coring device aligned with longitudinal myofiber orientation. Triplicate measurements were conducted per biological replicate, with peak shear force values (N/cm^2^) recorded during perpendicular cross-sectional blade penetration. Data were normalized to the cross-sectional area and expressed as mean ± SEM [20].

### 2.4. Determination of Chemical Composition of Muscles

Muscle moisture, crude protein, and ether extract were analyzed in duplicate according to AOAC methods [20]. Moisture was determined gravimetrically (oven drying at 105 °C). Crude protein and ether extract content were measured using the Kjeldahl and Soxhlet extraction methods, respectively [21].

### 2.5. Histological Analysis of Tissues

Muscle, rumen, duodenum, jejunum, and ileum tissues were fixed in 4% paraformaldehyde for 24 h and paraffin-embedded. Serial sections (5 µm thickness) were prepared and stained with hematoxylin and eosin (H&E) following established protocols [22]. Morphometric analysis of ileal villus length and crypt depth was performed using Image J1 software (National Institutes of Health, Bethesda, MD, USA).

### 2.6. Blood Sampling and Biochemical Analyses

Blood samples (10 mL) were collected via jugular venipuncture into sodium heparin-coated vacutainers (750 IU/mL) pre-slaughter. Plasma was isolated by centrifugation (3500× *g*, 10 min, and 4 °C), aliquoted into sterile 2 mL microcentrifuge tubes (Beijing North Institute of Biological Technology, Beijing, China), and stored at −80 °C until analysis. Antioxidant parameters (total antioxidant capacity [T-AOC], catalase [CAT], glutathione [GSH], and malondialdehyde [MDA]), lipid metabolites (triglycerides [TG], total cholesterol [T-CHO], low-density lipoprotein cholesterol [LDL-C], and high-density lipoprotein cholesterol [HDL-C]), and hepatic/renal biomarkers (blood urea nitrogen [BUN], creatinine [CRE], glutamic-oxaloacetic transaminase [GOT], glutamic-pyruvic transaminase [GPT], and alkaline phosphatase [AKP]) were quantified using commercial competitive ELISA kits (Nanjing Jiancheng Bioengineering Institute, Nanjing, China) per manufacturer protocols. Absorbance measurements were performed in triplicate using an ELx800™ microplate reader (BioTek Instruments, Winooski, VT, USA) [23].

### 2.7. Gastrointestinal Microbiota Analysis

Gastrointestinal content were aseptically collected postmortem and immediately stored at −80 °C for microbial profiling. Total microbial RNA was isolated and purified using an AllPrep^®^ PowerFecal^®^ Pro DNA/RNA Kit (QIAGEN, cat.2003504, Germantown, MD, USA) following the manufacturer’s procedure. Then, the total RNA were reverse-transcribed to create the cDNA by SuperScript™ II Reverse Transcriptase (Invitrogen, cat. 1896649, Carlsbad, CA, USA). Primers were designed according to conserved regions of 16S rDNA sequence, and one-step PCR was performed using reverse-transcribed microbial cDNA as a template. After 35 cycles of PCR, sequencing adapters and barcodes were added for amplification. PCR amplification products were detected by 1.5% agarose gel electrophoresis. The target fragments were recovered using the AxyPrep PCR Cleanup Kit (Shanghai Goldside Biotechnology Co., Shanghai, China). The PCR product was further purified using the Quant-iT PicoGreen dsDNA Assay Kit (Molecular Probes, Eugene, OR, USA). The library was quantified on the Promega QuantiFluor fluorescence quantification system (Promega Corporation, Madison, WI, USA). The pooled library was loaded on an Illumina platform using a paired-end sequencing protocol (2 × 250 bp) [16,24].

Sequencing primers were eliminated from demultiplexed raw sequences using Cutadapt (v1.9). Paired-end reads were merged with FLASH (v1.2.8). Low-quality reads (quality scores <20), short reads (<100 bp), and reads containing >5% ambiguous bases (“N”) were trimmed via the sliding-window algorithm implemented in fqtrim (v0.94). High-quality clean tags were obtained through quality filtering using fqtrim. Chimeric sequences were removed using Vsearch (v2.3.4). Denoising and generation of amplicon sequence variants (ASVs) were performed using DADA2. Taxonomic annotation of sequences was conducted via the QIIME2 plugin feature-classifier, referenced against the SILVA and NT-16S databases. Alpha and beta diversity metrics were computed in QIIME2. Bacterial taxonomic profiles were analyzed using relative abundance. Differentially abundant genera were identified via Wilcoxon rank-sum tests, with statistical significance defined as *p* < 0.05. Linear discriminant analysis effect size (LEfSe; LDA score ≥ 3.0, *p* < 0.05) was executed using the Segata LEfSe tool.

### 2.8. Statistical Analyses

All datasets (slaughter performance, meat quality, muscle biochemical composition, and blood biochemical indices) were analyzed using SPSS 25.0 (IBM Corp., Armonk, NY, USA) under a completely randomized design with the treatment as the fixed effect. One-way analysis of variance (ANOVA) with Bonferroni post hoc correction was conducted for omnibus comparisons, followed by unpaired two-tailed Student’s *t*-tests for pairwise group contrasts. Normality and homogeneity of variance assumptions were verified via Shapiro–Wilk and Levene’s tests, respectively. Spearman’s rank correlation analysis was performed to assess associations between gut microbiota modulation, antioxidant indices, muscle composition, and fat deposition. GraphPad Prism 7 (GraphPad Inc., San Diego, CA, USA) software was used for graphing, and data results were expressed as mean ± standard deviation, with *p* < 0.05 considered significant.

## 3. Results

### 3.1. Slaughter Performance

Melatonin implantation exhibited no significant effects (*p* > 0.05) on slaughter live weight, carcass weight, dressing percentage, or morphometric indices (body height, body length, chest circumference, tube circumference, and GR value) in cashmere goats. However, slaughter weight and carcass weight demonstrated a decreasing trend. Notably, melatonin administration reduced visceral adiposity, with perirenal fat (470.74 vs. 299.38 g), greater omental (946.86 vs. 569.20 g), and mesenteric fat (400.06 vs. 312.58 g) exhibiting marked decreases (*p* < 0.05; Table 2).

No significant differences (*p* > 0.05) were observed in absolute organ weights (heart, liver, spleen, lungs, kidneys, and rumen) or organ indices between groups, though organ indices for heart, liver, spleen, lungs, and kidneys displayed an upward tendency (Table 3).

### 3.2. Meat Quality and Muscle Composition

Melatonin administration improved meat quality parameters relative to controls (Table 4). In the LTL, melatonin treatment induced a nonsignificant elevation in pH_45_min but increased pH_24_h (5.71 vs. 6.26, *p* < 0.05). Melatonin reduced yellowness (b*, 4.62 vs. 3.96, *p* < 0.05), lowered shear force (12.88 vs. 9.72 kgf, *p* < 0.05), and increased CP (61.93% vs. 55.20%, *p* < 0.05), with concurrent trends toward reduced drip loss and elevated moisture (Table 4).

In the GL, no significant treatment effects were detected (*p* > 0.05), though trends emerged toward higher pH_45_min and pH_24_h, reduced drip loss and shear force, and elevated crude protein. For the BF, melatonin increased pH_45_min and pH_24_h (nonsignificant trends), reduced drip loss and shear force, and significantly elevated moisture and CP (*p* < 0.05) (Table 4).

**Figure 1:**
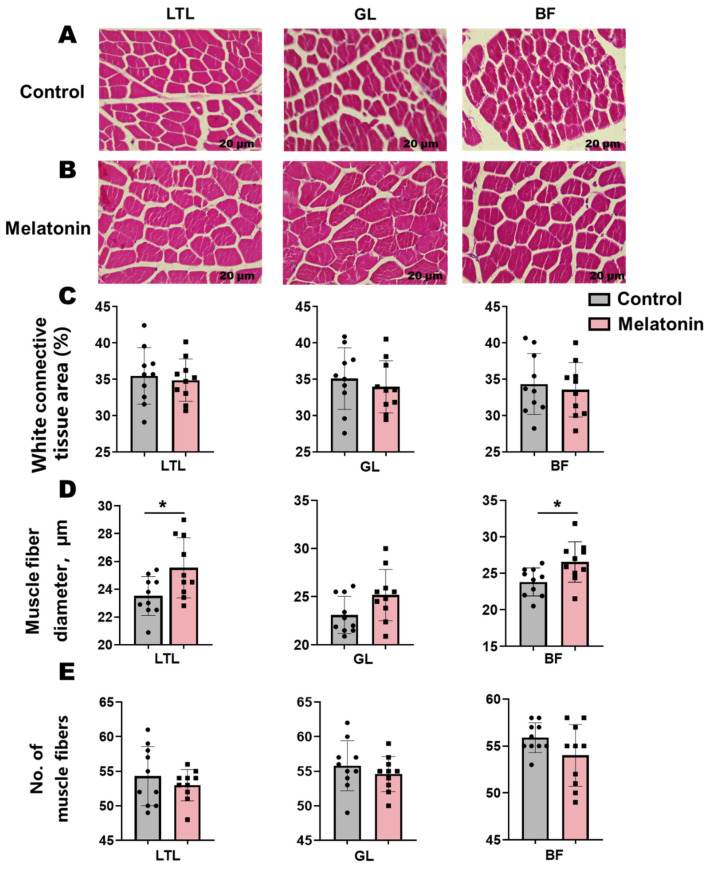
Histological analysis and morphometric parameters of skeletal muscles (*Longissimus thoracis et lumborum* (LTL), Gluteus (GL), and biceps femoris (BF)) in control versus melatonin-treated Liaoning cashmere goats. (**A**,**B**) Representative H&E-stained sections (scale bar = 20 µm). (**C**) White connective tissue area, (**D**) muscle fiber diameter, and (**E**) muscle fiber number across the LTL, GL, and BF muscles. * *p* < 0.01.

H&E staining of muscle sections revealed an elevation (*p* < 0.05) in muscle fiber diameter within the LTL and BF following melatonin treatment (Figure 1A,B). Concomitant reductions in connective tissue area and muscle fiber number were observed across these muscles, though these trends did not reach statistical significance (Figure 1C–E).

### 3.3. Blood Biochemical Parameters

**Figure 2:**
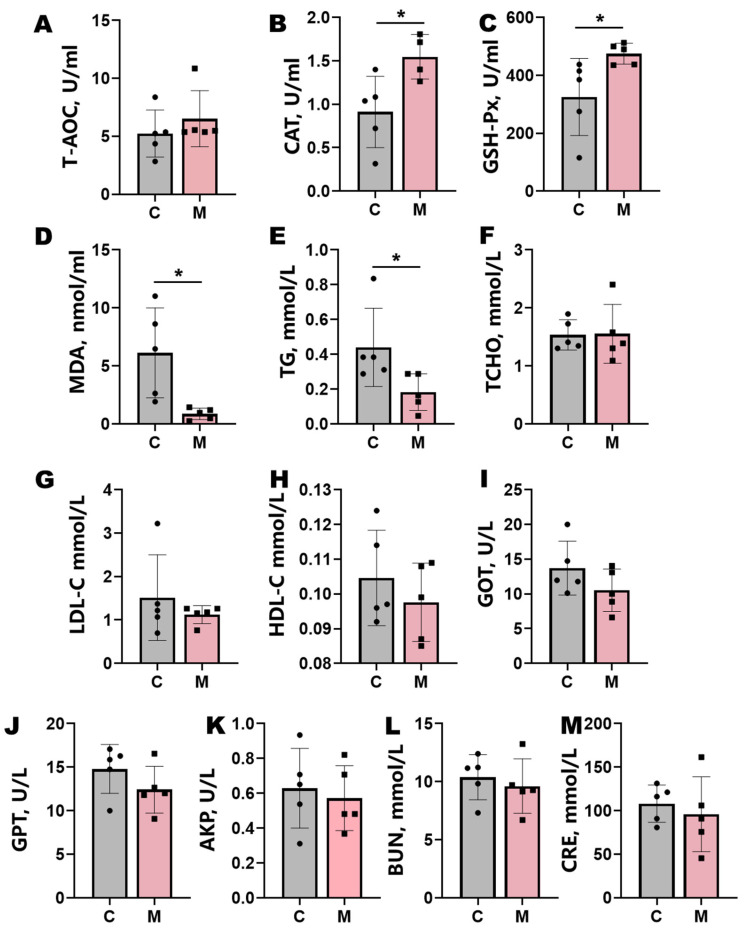
Blood biochemical parameters in control (**C**) and melatonin-treated (**M**) groups. (**A**–**D**) Antioxidant indices: total antioxidant capacity (T-AOC), catalase (CAT), glutathione peroxidase (GSH-Px), and malondialdehyde (MDA). (**E**–**H**) Lipid metabolism markers: triglyceride (TG), total cholesterol (TCHO), low-density lipoprotein cholesterol (LDL-C), and high-density lipoprotein cholesterol (HDL-C). (**I**–**M**) Liver and kidney function parameters: blood urea nitrogen (BUN), creatinine (CRE), glutamic oxaloacetic transaminase (GOT), glutamic pyruvic transaminase (GPT), and alkaline phosphatase (AKP). Data represent mean ± SD (*n* = 5); statistical significance (*t*-test): *p* < 0.05 and * *p* < 0.01.

Melatonin implantation significantly enhanced systemic antioxidant capacity in Liaoning cashmere goats (Figure 2). Treated animals exhibited marked elevations in plasma CAT and GSH-Px activities (*p* < 0.05), coupled with a substantial reduction in MDA levels (*p* < 0.05) (Figure 2A–D). Concomitant with these antioxidant effects, melatonin administration enhanced lipid metabolism, significantly lowering plasma TG levels (*p* < 0.05) and inducing nonsignificant downward trends in TCHO, LDL-C, and HDL-C (Figure 2E–H). Plasma CRE displayed a nonsignificant decreasing trend (*p* > 0.05). Hepatic biomarkers GOT, GPT, and AKP demonstrated attenuated activity trends following treatment, though these changes lacked statistical significance (*p* > 0.05; Figure 2I–M).

### 3.4. Gastrointestinal Histomorphology and Microbiome Composition

**Figure 3:**
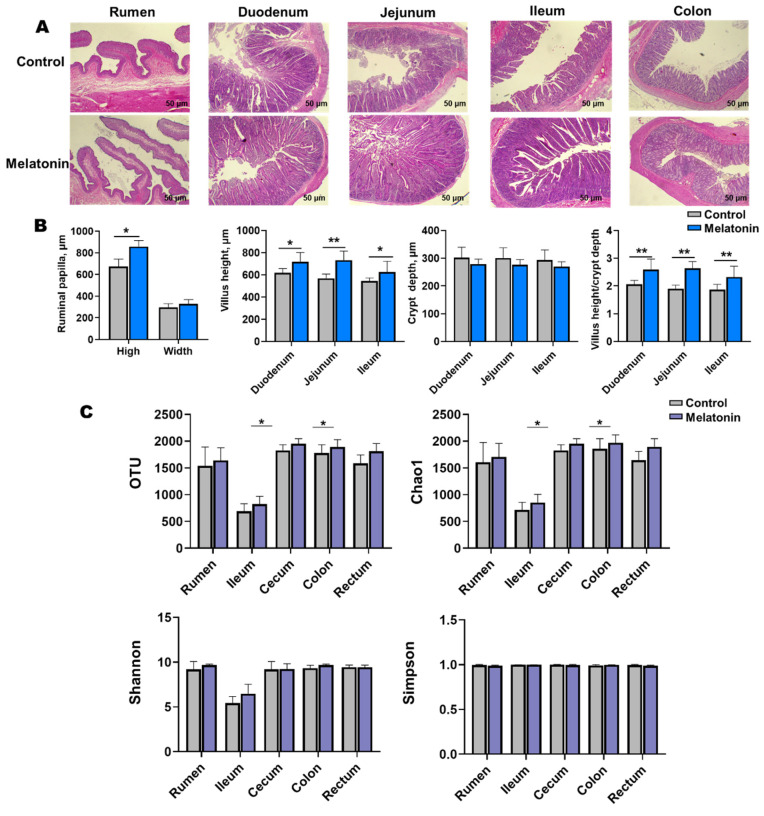
Gastrointestinal histology and microbial diversity control vs. melatonin-treated groups at slaughter. (**A**) Representative H&E-stained sections (scale bar = 50 µm) of rumen, duodenum, jejunum, ileum, and colon. (**B**) Villus height and crypt depth (statistical analysis). (**C**) Alpha diversity indices (OTUs, Chao1, Shannon, and Simpson) of microbial communities in rumen fluid, ileal, cecal, colonic, and rectal content. * *p* < 0.01 and ** *p* < 0.001.

Melatonin-treated goats exhibited enhanced morphological development in the rumen, duodenum, jejunum, and ileum (Figure 3A). Villus height-to-crypt depth ratios increased significantly (*p* < 0.05), with marked elevations in villus height observed in the duodenum, jejunum, ileum, and rumen. Rumen papillae height was also significantly greater in treated animals (*p* < 0.05, Figure 3B).

**Figure 4:**
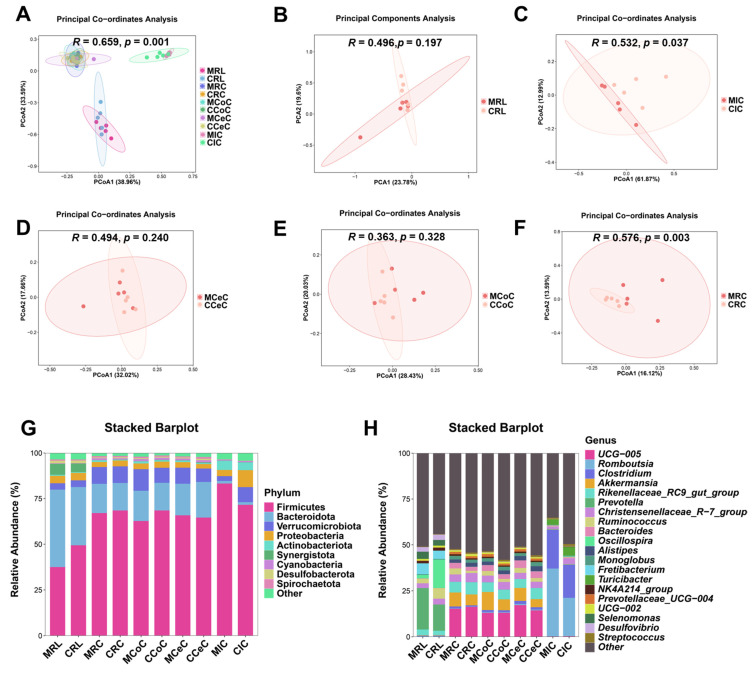
Beta diversity analysis and taxonomic composition of gastrointestinal microbiota in control and melatonin-group Liaoning cashmere goats. (**A**–**F**) PCoA of microbial communities (Bray-Curtis distances) in rumen fluid (**B**), ileal (**C**), cecal (**D**), colonic (**E**), and rectal (**F**) content. (**G**,**H**) Bacterial community structure at the (**G**) phylum and (**H**) genus levels.

Alpha diversity analysis revealed elevated microbial richness (observed OTUs, Chao1) in the cecal and rectal content of the melatonin group (*p* < 0.05), with similar trends in the rumen, ileum, and colon (Figure 3C). Simpson and Shannon indices showed no significant intergroup differences (*p* > 0.05) but trended higher in treated animals. Beta diversity (Bray-Curtis PCoA) demonstrated distinct clustering of ileal (*p* = 0.037, *R* = 0.532; Figure 4C) and rectal (*p* = 0.003, *R* = 0.576; Figure 4F) microbiota between two groups.

**Figure 5:**
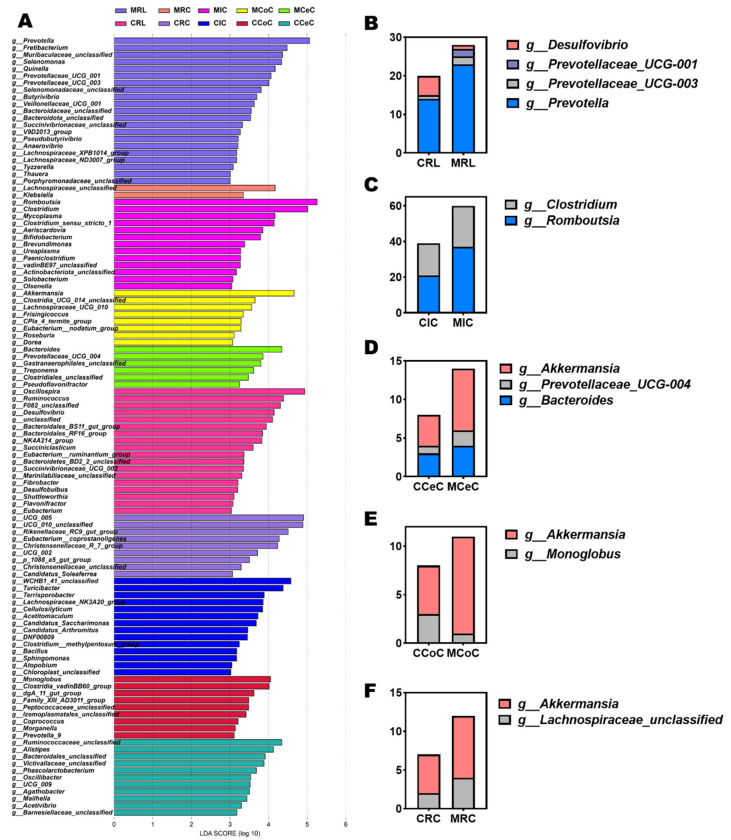
Differentially abundant microbial genera and LEfSe analysis of gastrointestinal microbiota in melatonin-treated Liaoning cashmere goats. (**A**) LEfSe analysis identifying genus-level differentially abundant taxa (LDA score > 3, *p* < 0.05) across gastrointestinal regions. (**B**–**F**) Group-specific, significantly differentially abundant genera in rumen fluid, ileal, cecal, colonic, and rectal digesta between control and melatonin-treated groups.

Dominant phyla across gastrointestinal regions included Firmicutes, Bacteroidota, Verrucomicrobiota, and Proteobacteria (Figure 4G). At the genus level, *Prevotella*, *Romboutsia*, *Clostridium*, *Akkermansia*, *Bacteroides*, and *Prevotellaceae_UCG-004* were prevalent (Figure 4H and Figure 5B–F). LEfSe analysis revealed melatonin-enriched discriminators (LDA score >3.0) and melatonin-increased ruminal *Prevotella* (14.28 vs. 22.50, *p* < 0.05), *Prevotellaceae_UCG-001*, and *Prevotellaceae_UCG-003* abundance while reducing *Desulfovibrio* (*p* < 0.05, Figure 5B). Ileal *Romboutsia* (20.85 vs. 36.87, *p* < 0.05) and Clostridium (18.01 vs. 21.73, *p* < 0.05) increased significantly (Figure 5C), whereas cecal *Prevotellaceae_UCG-004* and *Bacteroides* were elevated (*p* < 0.05; Figure 5D). Colonic *Monoglobus* abundance decreased (*p* < 0.05; Figure 5E). Notably, *Akkermansia* abundance rose significantly in cecal (4.41 vs. 7.04, *p* < 0.05), colonic (7.08 vs. 10.82, *p* < 0.05), and rectal (5.01 vs. 7.89, *p* < 0.05) content of melatonin-treated goats (Figure 5D–F).

**Figure 6:**
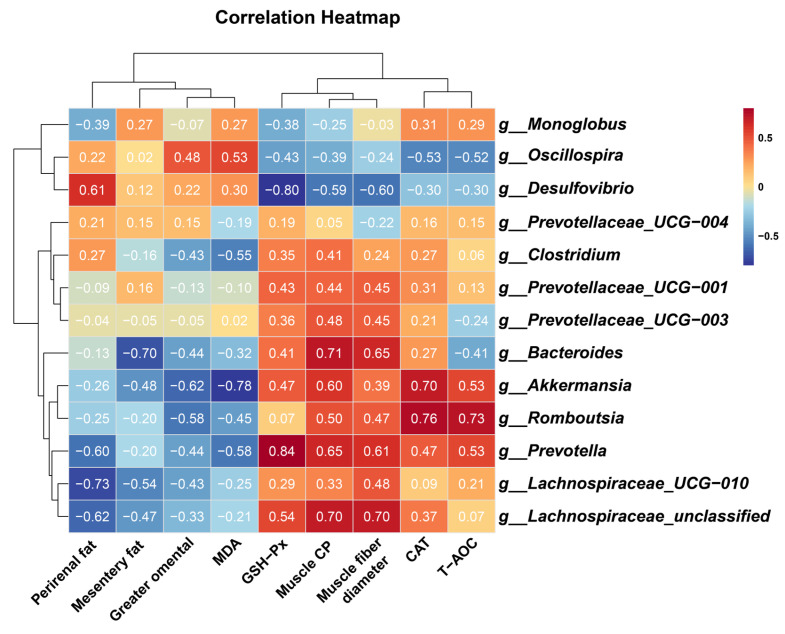
Heatmap of spearman correlation between phenotypic variables and microbial genus in control and melatonin-group cashmere goats. Muscle CP, crude protein content of longissimus thoracis et lumborum; muscle fiber diameter, muscle fiber diameter of longissimus thoracis et lumborum; T-AOC, total antioxidant capacity; CAT, catalase; GSH-Px, glutathione peroxidase; MDA, malondialdehyde. The numbers in the figure represent the correlation coefficients.

To elucidate potential mechanistic links between gut microbiota modulation, antioxidant enhancement, muscle growth, and fat deposition, Spearman’s rank correlation analysis was performed. Key microbial taxa exhibited significant associations with physiology and metabolic indices:

Beneficial taxa included the following: *g__Prevotella* demonstrated strong positive correlations with muscle CP (*R* = 0.648, *p* = 0.049), muscle fiber diameter (*R* = 0.612, *p* = 0.056), and GSH-PX activity (*R* = 0.839, *p* = 0.002). *g__Romboutsia* correlated positively with CAT activity (*R* = 0.758, *p* = 0.016) and T-AOC (*R* = 0.730, *p* = 0.017). *g__Akkermansia* showed inverse associations with MDA levels (*R* = −0.782, *p* = 0.012) and greater omental fat deposition (*R* = −0.624, *p* = 0.050), alongside positive correlations with CAT activity (*R* = 0.697, *p* = 0.031). *g__Lachnospiraceae_UCG-010* and *g__Lachnospiraceae_unclassified* were inversely linked to perirenal fat (*R* = −0.733, *p* = 0.021) and positively associated with muscle CP (*R* = 0.697, *p* = 0.031) and muscle fiber diameter (*R* = 0.697, *p* = 0.031). Adverse taxa included the following: *g__Desulfovibrio* displayed negative correlations with GSH-PX activity (*R* = −0.796, *p* = 0.006), muscle CP (*R* = −0.588, *p* = 0.080), and muscle fiber diameter (*R* = −0.600, *p* = 0.073), while showing positive trends with perirenal fat accumulation (*R* = 0.612, *p* = 0.066) (Figure 6).

## 4. Discussion

Slaughter performance serves as a critical metric for evaluating livestock production efficiency and economic viability. In this study, melatonin implantation elicited no significant alterations (*p* > 0.05) in slaughter metrics of 8-month-old cashmere goats, though a downward trend in body and carcass weights was observed. These findings align with prior reports demonstrating that melatonin administration (2 mg/kg) in Inner Mongolian cashmere goats induced non-significant reductions in daily weight gain [25]. Our previous investigations corroborate that melatonin does not significantly influence growth performance or nutrient digestibility in this species but enhances systemic antioxidant capacity and nitrogen retention efficiency [2].

Excessive visceral adipogenesis in livestock represents a metabolic inefficiency, increasing feed conversion ratios and diminishing production profitability. Notably, melatonin treatment significantly reduced perirenal, greater omental, and mesenteric adipose tissue mass in this study. This anti-lipogenic effect parallels observations in murine and human models, where melatonin modulates insulin sensitivity, suppresses nocturnal glucose tolerance, and inhibits hypertrophic adipocyte expansion [26]. Mechanistically, melatonin attenuates the diet-induced dysregulation of redox enzyme gene expression in rodent subcutaneous and perirenal adipose depots. Furthermore, it regulates the circadian transcription of lipolytic regulators (*Cgi58*, *Perilipin*, and *Dgat1*) in mesenteric adipose tissue, stabilizing free fatty acid flux during dark-phase metabolism in obese rats [27]. These findings suggest melatonin may enhance energy partitioning by modulating insulin signaling pathways, preferentially reducing visceral fat accretion without compromising lean mass. This selective lipid-lowering effect, coupled with unchanged body weight, positions melatonin as a potential metabolic modifier to optimize feed efficiency in cashmere goat.

Muscle fiber properties fundamentally govern both quality and yield attributes of meat [28]. In this study, melatonin administration significantly reduced drip loss, significantly increased myofiber diameter, significantly elevated CP, stabilized postmortem PH, and significantly increased the body’s antioxidant capacity. These effects align with melatonin’s documented capacity to mitigate oxidative muscle damage via free radical scavenging and upregulation of antioxidant enzymes (superoxide dismutase and glutathione peroxidase) in both physiological and pathological states [29,30]. The differences in the three muscle-type quality parameters reflects divergent fiber-type composition and metabolic programming. The oxidative fiber-dominant LTL exhibited delayed pH decline due to prolonged mitochondrial ATP synthesis, reducing proteolysis and drip loss [31]. Conversely, glycolytic BF demonstrated accelerated postmortem glycolysis, correlating with heightened protein turnover and lower CP retention [32]. Melatonin’s tissue-specific effects likely stem from its preferential modulation of oxidative metabolism, evidenced by mitochondrial biogenesis and inhibition of ROS-mediated apoptosis in LTL [33]. Notably, melatonin preserves mitochondrial structural and functional integrity, attenuating age-associated myofiber atrophy and counteracting LPS-induced apoptosis via the TNFRSF12A/caspase-8 axis in sarcopenia models [33,34]. At the transcriptional level, melatonin potentiates myogenesis by upregulating the following: (1) *PAX7* (satellite cell activation), (2) MYOG (myoblast fusion commitment), (3) myosin heavy-chain isoforms (*MYHC IIA* and *MYHC IIB*), and (4) IGF-1/IGFBP5 axis components in LTL muscle [9]. Concurrently, melatonin amplifies anabolic hormone signaling (growth hormone and testosterone) and modulates apoptotic pathways in myocytes, leading to hypertrophic fiber growth [8]. These pleiotropic melatonin mechanisms, spanning redox regulation, transcriptional control, and endocrine modulation, collectively enhance muscle fiber properties and meat quality.

Compelling evidence establishes the gut microbiome as a master regulator of host protein deposition and lipid homeostasis through microbiota-derived metabolites (e.g., SCFAs and BCAAs) and endocrine signaling [35,36]. Melatonin administration significantly enhanced gastrointestinal histomorphology and microbial α-diversity in cashmere goats, with marked increases in the relative abundances of *Prevotella*, *Romboutsia*, *Clostridium*, and *Akkermansia*, and significantly decreased the relative abundances of *Desulfovibrio* across gastrointestinal tract. *Prevotella*, a dominant rumen symbiont, facilitates plant polysaccharide hydrolysis and nitrogen metabolism, driving volatile fatty acid (VFA) production critical for rumen function and energy supply [37,38]. Myostatin-knockout (MSTN-KO) cattle exhibit elevated *Prevotella* abundance concomitant with increased muscle mass and branched-chain amino acid (BCAA) levels, suggesting its role in myogenesis via enhanced BCAA biosynthesis [39]. Similarly, *Clostridium butyricum* supplementation amplifies ruminal *Prevotella* populations, activating the IGF-1/Akt/mTOR signaling axis to stimulate protein synthesis and hypertrophic fiber growth [40].

*Romboutsia* regulates gut metabolism and immunity, crucial for maintaining intestinal homeostasis and health [41,42]. Dietary fermented sweet potato residue (FSPR) supplementation elevated cecal *Romboutsia* abundance (2.0-fold), enhanced crude protein digestibility, and improved slaughter performance and breast muscle water-holding capacity in broilers [43]. Increased feeding frequency (four meals vs. two) alters cecal microbiota (higher Clostridium and *Romboutsia*) and bile acid profiles (elevated primary, reduced taurine-conjugated bile acids), improves feed efficiency, and enhances muscle growth (via slow-twitch fiber conversion and myogenesis) in finishing pigs [44]. While *Romboutsia* has been primarily studied in monogastric species, emerging evidence highlights its role in ruminant muscle metabolism. For instance, a recent ontogenetic analysis of Tibetan sheep (Ovis aries) revealed age-dependent shifts in ileal *Romboutsia* abundance, which correlated positively with beneficial fatty acids (e.g., oleic acid and C18:1n9c) in the LTL and BF muscles. This aligns with our findings, where melatonin-induced enrichment of *Romboutsia* in cashmere goats coincided with elevated intramuscular protein content [45].

*Akkermansia muciniphila*, a mucin-degrading symbiont, critically maintains intestinal health by fortifying the mucus layer, modulating TLR signaling, and producing immunoregulatory metabolites [46,47]. Dietary interventions amplifying *Akkermansia* and *Lachnoclostridium* (3.5- and 2.1-fold, respectively) abundances in swine upregulate lipid/amino acid metabolic pathways, influencing both growth performance and meat flavor profiles [48]. Lambs’ dietary L-arginine (ARG) supplementation significantly increased the abundance of beneficial gut microbiota (*Akkermansia*), promoted the deposition of nutritionally favorable fatty acids (FAs) in the LTL muscle, and modulated FA profiles in subcutaneous adipose tissue (SAT), suggesting a microbiota-driven mechanism for improving lipid quality in muscle and adipose depots mediated by ARG [49]. Accumulating evidence elucidates a mechanistic link between *Desulfovibrio* enrichment and adipogenic as well as carcinogenic pathways, with recent studies demonstrating that high-fat dietary regimes induce gut dysbiosis marked by pronounced *Desulfovibrio* proliferation [50,51]. These findings collectively position melatonin as a microbiota-modulating agent capable of optimizing nutrient partitioning and muscle protein deposition in cashmere goats.

## 5. Conclusions

Melatonin supplementation in Liaoning cashmere goats enhances muscle growth and reduces visceral adiposity by remodeling gut microbiota and enhancing antioxidant capacity. Melatonin-treated animals exhibited muscle fiber hypertrophy, reduced fat depots, improved meat quality (elevated pH, protein content, and antioxidant capacity), and suppressed drip loss. Melatonin enriched beneficial genera (*Prevotella*, *Romboutsia*, and *Akkermansia*), inhibited lipogenic *Desulfovibrio*, and enhanced intestinal villus–crypt ratios and α-diversity. These microbiota-driven shifts optimized nutrient partitioning, favoring protein anabolism over lipid deposition. The findings highlight melatonin as a dual-action regulator for muscle–fat balance via gut–microbe crosstalk, offering a sustainable strategy to enhance meat yield and quality in ruminants. Future studies should include males to evaluate sex-specific responses and conduct systematic dose-response analyses to optimize melatonin regimens for muscle–fat modulation in cashmere goats.

## Figures and Tables

**Table 1:** Ingredients and nutritional level of basal diets (DM basis).

Ingredient	(%)	Nutrient Levels ^2^	
Alfafa	35.00	ME, (MJ/Kg)	9.69
Peanut straw	35.00	DM, (%)	86.99
Corn	16.00	EE, (%)	3.62
Soybean meal	9.50	CP, (%)	14.80
Fermented soybean meal	3.50	Ca, (%)	1.00
Dicalcium phosphate	0.10	P, (%)	0.32
Salt	0.50	NDF, (%)	24.66
Premix ^1^	0.30	ADF, (%)	47.38
Total	100.00	ASH, (%)	6.70

^1^ Premix: FeSO_4_·7H_2_O 170 g/kg; CuSO_4_·5H_2_O 70 g/kg; MnSO_4_·5H_2_O 290 g/kg; ZnSO_4_·7H_2_O 240 g/kg; CoCl_2_·6H_2_O 510 mg/kg; KI 220 mg/kg; Na_2_SeO_3_ 130 mg/kg; VA 1, 620,000 IU/kg; VD3 324,000 IU/kg; VE 540 IU/kg; VK3 150 mg/kg; VB1 60 mg/kg; VB2 450 mg/kg; VB12 0.9 mg/kg. ^2^ Nutrients are measured values except for metabolizable energy, which is calculated. ME, metabolizable energy; DM, dry matter; CP, crude protein; EE, ether extract; ADF, acid detergent fiber; NDF, neutral detergent fiber.

**Table 2:** Effects of melatonin supplementation on carcass traits and visceral fat deposition in Liaoning cashmere goats.

Item	C	M	*p*-Value
Slaughter weight (kg)	28.73 ± 0.84	26.26 ± 0.65	0.071
Carcass weight (kg)	12.40 ± 0.48	11.27 ± 0.36	0.073
Carcass yield (%)	43.13 ± 0.73	43.05 ± 1.07	0.958
Height at withers (cm)	52.55 ± 0.96	50.01 ± 0.80	0.106
Length of body (cm)	56.30 ± 0.48	54.83 ± 0.95	0.245
Circumference of chest (cm)	8.98 ± 0.12	8.64 ± 0.15	0.163
Head weight (kg)	1.79 ± 0.04	1.74 ± 0.02	0.215
Hoof weight (kg)	0.70 ± 0.02	0.68 ± 0.02	0.158
GR (cm)	4.82 ± 0.08	4.18 ± 0.16	0.322
Rib-eye area (cm^2^)	20.41 ± 2.35	18.33 ± 0.98	0.182
Perirenal fat (g)	470.74 ± 43.56 ^a^	299.38 ± 49.70 ^b^	0.049
Greater omental (g)	946.86 ± 48.52 ^a^	569.20 ± 58.06 ^b^	0.002
Mesentery fat (g)	400.06 ± 41.47 ^a^	312.58 ± 23.07 ^b^	0.044

C, control group; M, melatonin group. Data are presented as mean ± SEM (*n* = 5). Statistical significance (*p* < 0.05) was determined via unpaired two-tailed Student’s *t*-test. Dissimilar superscript letters denote significant intergroup differences.

**Table 3:** Effects of melatonin on visceral organ weight and index of Liaoning cashmere goats.

Item		C	M	*p*-Value
Weight (g)	Heart	78.04 ± 4.16	68.94 ± 1.73	0.108
	Liver	467.20 ± 39.23	428.38 ± 14.88	0.432
	Spleen	30.62 ± 0.94	28.84 ± 2.13	0.514
	Lung	265.74 ± 21.78	219.92 ± 13.77	0.972
	Kidney	82.62 ± 2.28	70.36 ± 2.31	0.100
Index (%)	Heart	0.27 ± 0.01	0.29 ± 0.01	0.139
	Liver	1.62 ± 0.10	1.81 ± 0.08	0.177
	Spleen	0.11 ± 0.01	0.12 ± 0.01	0.172
	Lung	0.93 ± 0.08	0.93 ± 0.07	0.972
	Kidney	0.29 ± 0.01	0.24 ± 0.01	0.422
Ovarian (g)		1.56 ± 0.15	1.55 ± 0.14	0.997
Rumen weight (kg)		3.60 ± 0.47	3.16 ± 0.31	0.499
Net rumen (kg)		1.07 ± 0.12	1.05 ± 0.11	0.932
Rumen pH		6.74 ± 0.26	6.79 ± 0.09	0.904

C, control group; M, melatonin group. Data are presented as mean ± SEM (*n* = 5). Statistical significance (*p* < 0.05) was determined via unpaired two-tailed Student’s *t*-test.

**Table 4:** Effects of melatonin administration on meat quality and muscle composition in Liaoning cashmere goats.

Item		C	M	*p*-Value
LTL	pH_45_min	6.12 ± 0.20	6.44 ± 0.23	0.076
	pH_24_h	5.71 ± 0.23 ^b^	6.26 ± 0.36 ^a^	0.033
	L*	37.76 ± 1.66	38.07 ± 1.54	0.795
	a*	19.32 ± 1.33	17.82 ± 1.43	0.164
	b*	4.62 ± 0.44 ^a^	3.96 ± 0.34 ^b^	0.038
	Drip loss (%)	6.47 ± 2.79	2.98 ± 1.86	0.071
	Cooking loss (%)	50.54 ± 4.85	43.35 ± 6.68	0.129
	Shear force (kgf)	9.72 ± 3.34 ^b^	12.88 ± 2.00 ^a^	0.008
	Moisture content (%)	66.90 ± 2.43	71.54 ± 0.57	0.100
	Crude protein content (%)	55.20 ± 2.44 ^b^	61.93 ± 0.99 ^a^	0.034
	Ether extract content (%)	8.84 ± 1.37	7.80 ± 1.15	0.555
GL	pH_45_min	6.39 ± 0.25	6.61 ± 0.08	0.133
	pH_24_h	5.69 ± 0.29	6.01 ± 0.27	0.178
	L*	36.15 ± 1.30	36.97 ± 0.98	0.509
	a*	18.88 ± 0.91	17.80 ± 1.41	0.822
	b*	4.02 ± 0.44	4.05 ± 0.46	0.676
	Drip loss (%)	4.52 ± 2.10	3.10 ± 1.94	0.351
	Cooking loss (%)	53.25 ± 4.12	50.04 ± 6.97	0.451
	Shear force (kgf)	9.28 ± 4.35	12.59 ± 2.10	0.208
	Moisture content (%)	70.75 ± 0.42	71.17 ± 0.70	0.567
	Crude protein content (%)	59.65 ± 4.60	64.25 ± 2.65	0.127
	Ether extract content (%)	9.46 ± 0.48	9.94 ± 1.85	0.802
BF	pH_45_min	6.34 ± 0.22	6.45 ± 0.14	0.339
	pH_24_h	5.75 ± 0.10	6.17 ± 0.43	0.093
	L*	37.76 ± 1.66	38.07 ± 1.54	0.343
	a*	19.32 ± 1.33	17.82 ± 1.43	0.237
	b*	4.65 ± 0.44	3.96 ± 0.34	0.930
	Drip loss (%)	3.42 ± 1.18	2.99 ± 1.63	0.679
	Cooking loss (%)	49.61 ± 5.38	45.20 ± 8.07	0.390
	Shear force (kgf)	6.60 ± 3.45	9.90 ± 0.95	0.102
	Moisture content (%)	69.70 ± 2.62 ^b^	72.32 ± 0.58 ^a^	0.003
	Crude protein content (%)	63.77 ± 4.91 ^b^	68.68 ± 0.98 ^a^	0.002
	Ether extract content (%)	7.77 ± 0.77	6.99 ± 0.98	0.451

C, control group; M, melatonin group. Data are presented as mean ± SEM (*n* = 5). Statistical significance (*p* < 0.05) was determined via unpaired two-tailed Student’s *t*-test. Dissimilar superscript letters denote significant intergroup differences. LTL, *longissimus thoracis et lumborum*. GL, Gluteus. BF, biceps femoris.

## Data Availability

All raw data and sequencing information can be requested by contacting the corresponding author Wei Zhang (wzhang@cau.edu.cn).
